# Behavioral disturbance and treatment strategies in Smith-Magenis syndrome

**DOI:** 10.1186/s13023-015-0330-x

**Published:** 2015-09-04

**Authors:** Alice Poisson, Alain Nicolas, Pierre Cochat, Damien Sanlaville, Caroline Rigard, Hélène de Leersnyder, Patricia Franco, Vincent Des Portes, Patrick Edery, Caroline Demily

**Affiliations:** Center for Screening and Treatment of Psychiatric Disorders of Genetic Origin, Vinatier Hospital, 95 Bd Pinel, 69678 Lyon, France; Cognitive Neuroscience Center, UMR 5229, French National Research Center (CNRS), Bron, France; Lyon 1 University, Lyon, France; Michel Jouvet Unite (sleep Medicine), Vinatier Hospital, Human chronobiology team INSERM 846, Bron, France; Pediatric Nephrology and Rhumatology Ward, Reference Center for Rare Kidney Diseases, Civil Hospices of Lyon, INSERM U820, Bron, France; Department of Genetics, Reference Center for Developmental Anomalies and Malformation Syndromes, Civil Hospices of Lyon, Bron, France; Hypnology Unit, Neuropediatric Ward, Civil Hospices of Lyon and INSERM U628, Lyon, France; Neuroscience Research Center of Lyon, Inserm U1028, CNRS UMR 5292, UCBL, TIGER Team, Bron, France; Pediatric Neurology Ward, Reference Center “Intellectual Deficiencies with Rare Causes”, Civil Hospices of Lyon, Bron, France. CNRS UMR 5304, L2C2, Institute of Cognitive Sciences, 69675 Bron, France

## Abstract

**Background:**

Smith-Magenis syndrome is a complex neurodevelopmental disorder that includes intellectual deficiency, speech delay, behavioral disturbance and typical sleep disorders. Ninety percent of the cases are due to a 17p11.2 deletion encompassing the *RAI1* gene; other cases are linked to mutations of the same gene. Behavioral disorders often include outbursts, attention deficit/hyperactivity disorders, self-injury with onychotillomania and polyembolokoilamania (insertion of objects into body orifices), etc. Interestingly, the stronger the speech delay and sleep disorders, the more severe the behavioral issues. Sleep disturbances associate excessive daytime sleepiness with nighttime agitation. They are underpinned by an inversion of the melatonin secretion cycle. However, the combined intake of beta-blockers in the morning and melatonin in the evening may radically alleviate the circadian rhythm problems.

**Discussion:**

Once sleep disorders are treated, the next challenge is finding an effective treatment for the remaining behavioral problems. Unfortunately, there is a lack of objective guidelines. A comprehensive evaluation of such disorders should include sleep disorders, potential causes of pain, neurocognitive level and environment (i.e. family and school). In any case, efforts should focus on improving communication skills, identifying and treating attention deficit/hyperactivity, aggressiveness and anxiety.

**Summary:**

Treatment of Smith-Magenis syndrome is complex and requires a multidisciplinary team including, among others, geneticists, psychiatrists, neuropediatricians/neurologists, somnologists, developmental and behavioral pediatricians, and speech and language therapists.

## Background

The treatment of genetic disorders associated with neurobehavioral phenotype is a major yet complex problem. Smith-Magenis syndrome (SMS) is one in many examples of this complexity. SMS is linked to a microdeletion of chromosome 17 in 90 % of the cases, and entails major behavioral disorders that jeopardize the social outcomes of the patients [1–4]. Its prevalence is estimated at 1 in 25,000, although this data may be an underestimation [5].

SMS is usually caused by a deletion of about 3.5 Mb on chromosome 17p11.2, and does not result from parental imprinting. The critical region includes the *RAI1* (Retinoic Acid Induced 1) gene and is less than 650 Kb in size [1–4, 6, 7]. Other genes potentially involved in the phenotype include: *PMP22*, which is linked to certain hereditary neuropathies of the Charcot Marie Tooth type (CMT) or hereditary neuropathy with liability to pressure palsy (HNPP); *TNFRSF13B*, which may cause immunodeficiency related to IgA deficiency; and *MYO15A*, which may cause hypoacousia [8–10]. These genes may account for the variability and severity of the phenotype, whereas the core symptoms seem to be linked to the haploinsufficiency of the *RAI1* gene [8, 11].

In general, the 17p11.2 deletion results from chromosome recombination errors during meiosis (crossing-over) favored by the repetition of certain genome sequences (LCR or low copy repeat) *via* a non-allelic homologous recombination mechanism (NAHR) [12]. These unequal meiotic recombinations are responsible for 70 % of SMS deletions [13]. LCR also favors the occurrence of reciprocal duplications, although these are far less often observed than deletions. Duplications in the 17p11.2 region lead to a different clinical picture, known as the Potocki-Lupski Syndrome (PTLS-OMIM 610883), which may be accompanied by autistic-spectrum disorders with hyperactivity, intellectual deficiencies, and congenital malformations.

In 10 % of the cases, the SMS phenotype results from a point mutation of *RAI1* in the heterozygous state. This gene's loss of function causes *RAI1* haploinsufficiency, which in turn results in a phenotype comparable to that of SMS by deletion. The *RAI1* gene is mainly expressed in brain tissue [14]. SMS diagnosis is established by examining a simple peripheral blood sample using fluorescence *in situ* hybridization (FISH) or comparative genomic hybridization (CGH array or ACPA), which is more costly than FISH but may enable the detection of an atypical deletion [4, 15]. If test results are negative and symptoms seem to point to SMS, a molecular biology study of the *RAI1* gene should be undertaken. Once the genetic anomaly (deletion or mutation) is identified in a proband, both parents should be tested to confirm a *de novo* event [15].

**Fig. 1:**
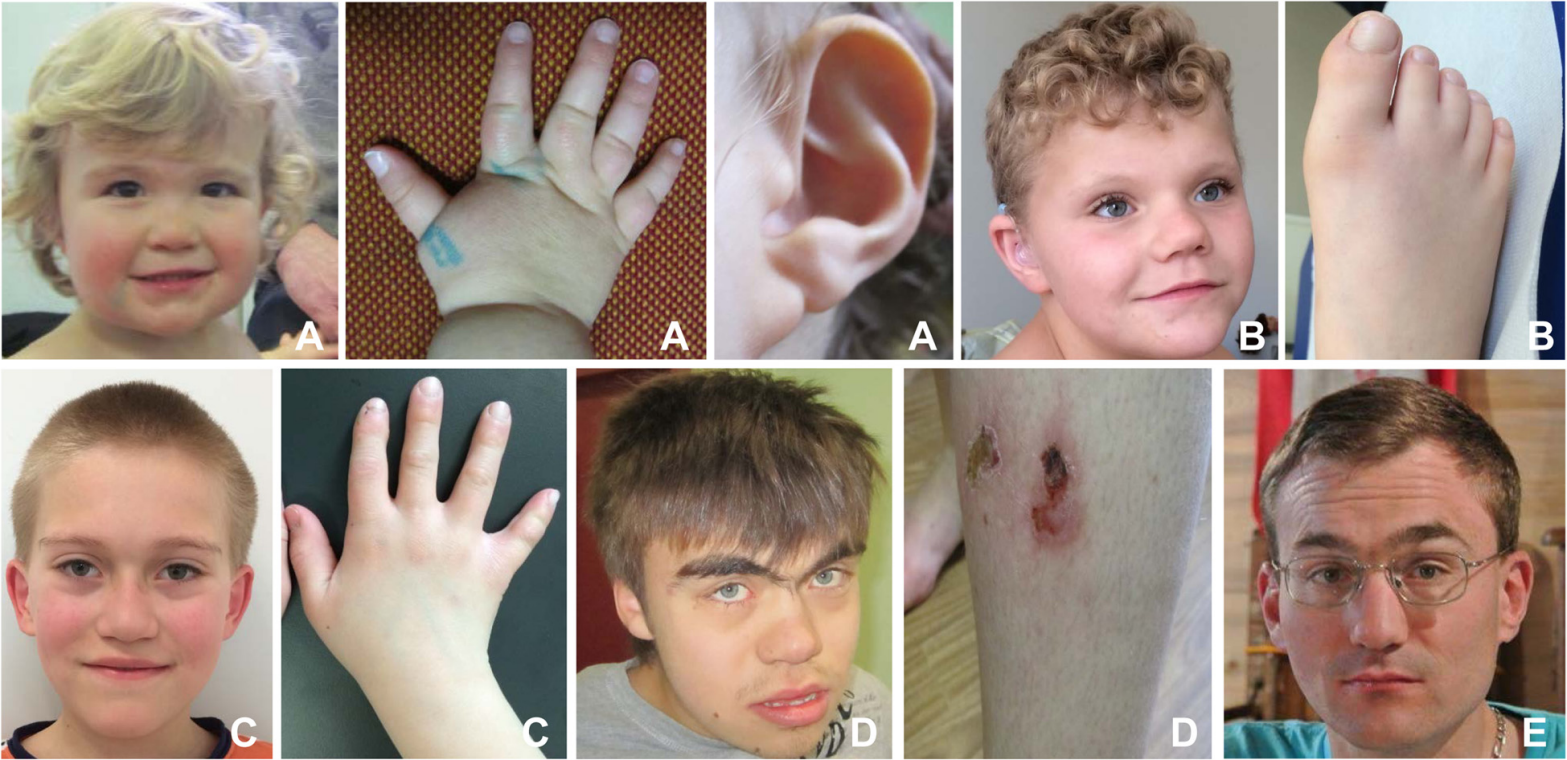
Typical SMS phenotype with ‘tented’ upper lip and depressed nasal bridge **a**, **b**, **c**, **d**, brachydactyly **a**, **b**. Young adults SMS often present with synophris (**d**, **e**) and prognatism **d**. Wounds from skin picking can be seen at any age **d**

SMS involves distinctive facial dysmorphia (Fig. 1). Children with SMS often have light-colored hair, bulging forehead, moderate hypoplasia of the middle part of the face and nasal bridge, hypertelorism, oblique outer and upper palpebral fissures, and synophrys. Micrognathia — readily observed during the first years of life — reverses over time, tending towards prognathism characterized by a wide, square-shaped face (Fig. 1). It may be associated with dental anomalies such as tooth agenesis (especially premolar teeth), and/or taurodontism. The child should be tested for ogival palate, short velum, and velopharyngeal insufficiency, especially before a general anesthetic is administered. Other common ear-nose-throat conditions include recurring ear infections, sometimes involving complications such as cholesteatoma, varying degrees of hypoacousia in 60 % of the cases (one third of which lead to perceptive deafness, two thirds to conduction deafness), hoarse voice, and vocal-cord nodes [7, 6–18].

About two thirds of young patients are of short stature. The limbs are short, with brachydactyly (Fig. 1). Some cases also show limited elbow-extension span, clinodactyly of the fifth finger, syndactyly of the second and third toes, persistence of fetal pads, and polydactyly. Scoliosis is frequent and should be systematically screened *via* clinical examination and spinal cord X-rays. Android obesity often appears in adolescence [2, 19–21]. Regarding the cardiovascular system, congenital heart disease has been reported for 30 % of the subjects, including interventricular or interatrial communication; tricuspid, mitral, pulmonary, or aortic stenosis; mitral valve insufficiency and/or prolapse; Fallot's tetralogy; and anomalous pulmonary venous return. Abdominal ultrasound may reveal spleen malformations but also developmental abnormalities in the kidneys and urinary system (unilateral or bilateral renal hypodysplasia, renal ectopy and agenesis, ureter duplicity, hydronephrosis, and megaureter) [6, 22]. From an ophthalmological point of view, iris anomalies (heterochromia or hamartomas), strabismus, and microcornea have been reported. Refraction abnormalities are often found and frequently linked to hypermetropia. Retinal detachment has been noted, often trauma-related [23, 24]. The phenotype may vary among subjects presenting identical deletions or mutations, and even between monozygotic twins with SMS. This shows the absence of a simple correlation between genotype and phenotype [25, 26]. Hypothyroidism and hypercholesterolemia may be present, and these parameters should be tested regularly. Similarly, deficiencies in immunoglobulins A, E, and/or G may exist [20, 27].

In addition to the spectrum of physical differences there are also neuropsychological features of speech and language delay, sleep disruption, and behavioral disorders which need a comprehensive approach. With appropriate treatment, sleep can return to a normal cycle and behavioral disorders can be alleviated, thereby improving the well-being of the patients. Unfortunately, residual maladaptive behavior often persists despite the treatment of sleep disturbances, but there is a lack of objective guidelines. We propose below a comprehensive evaluation of behavioral disorders from symptoms to the patient’s environment. We suggest that the effective treatment of behavioral disorders in SMS is not limited to psychotropic drugs and should take into account the different steps of the evaluation.

## Discussion

### Neurological and developmental disorders in SMS

#### Sleep-wake rhythm disturbances

In the initial descriptions of SMS, the emphasis was mainly on maladaptive behavior and hyperactivity; sleep disorders were seldom mentioned [1, 2, 28]. One of the first studies focusing on sleep disturbances reported that 62 % of SMS persons presented with sleep disorders: difficulty falling asleep, problems staying asleep and frequent awakenings at night [6]. A total absence of paradoxical sleep (i.e. REM sleep) was sometimes observed [28]. Since then, several studies have explored the sleep patterns of SMS persons and confirmed previous data. They also introduced the notion of abnormal chronology of the light–dark cycle, which includes falling asleep and waking up early, and the need for several daytime naps [20, 29–31].

Sleep disorders in neurodevelopmental disorders are usually multi-factorial and not well understood. Interestingly, de Leersnyder and Potocki found a general perturbation of the sleep-wake rhythm in SMS, with inverted secretion of melatonin [30, 31]. Melatonin is the main hormone produced by the pineal gland from 5-hydroxytryptamine (5-HT). Normally, peak secretion by the pineal gland occurs in the middle of the night. It has been shown, dosing plasma melatonin and urinary metabolites that almost all SMS patients had a phase shift of their circadian rhythm of melatonin [30, 31]. Time at onset of melatonin secretion was around 6 AM and peak time was around 12 PM with a melatonin offset around 8 PM [30]. This observation led to an effective treatment of SMS disruptive sleep disorder that is detailed below. The synthesis of the melatonin is triggered by luminosity variations, i.e., it is inhibited by light. This light-driven system starts at the retina and then follows the retinohypothalamic tract to reach the suprachiasmatic nuclei of the hypothalamus. These nuclei are the seat of the main biological clock of mammals and are responsible for generating the organism's circadian rhythms. Several clock genes have been described. They control all circadian rhythms driven by environmental stimuli [32]. The expression of these genes oscillates at a circadian rhythm of approximately 24 h [32]. In SMS, there is only residual secretion of melatonin at night and an abnormal secretion peak around noon [30, 31]. We can assume, then, that a dysfunctional clock gene accounts for the sleep-wake circadian rhythm disorders in persons with SMS.

Recently, point mutations of the *RAI1* gene have been identified in persons presenting the clinical features of SMS with inversion of the melatonin secretion rhythm [33, 34]. These findings clearly stress the role of *RAI1* in SMS sleep disorders. Nevertheless, we know little about the mechanisms that account for the inverted circadian rhythm of melatonin secretion observed in SMS. In particular, the precise role of the *RAI1* in modulating light effects on sleep-wake rhythm remains unanswered.

The SMS sleep disturbance is likely multifactorial and inversion of melatonin secretion, clock genes disturbance, phase delay, and behavioral insomnia may contribute to sleep disturbance.

## Neurological disorders

An isolated decrease in active fetal movements is found in 50 % of SMS cases [35]. During the neonatal period, hypotonia and difficulty breast-feeding are often observed. These children are usually described by their parents as being very calm and sleeping a lot. Compared to other children, they seem to make fewer spontaneous movements and frequently show hypotonia, which may contribute to worsen their motor delay [36]. Their walk may be somewhat unstable but they do not present with true ataxia. SMS subjects seem to show a certain degree of insensitivity to pain, which may favor self-mutilation [37]. Concurrently, hyporeflexia is frequent but generally not accompanied by reduced motor or sensory conduction velocity. Certain persons with a large deletion that includes the *PMP22* gene may nevertheless present with HNPP [20, 35]. Some patients (10-30 %) develop epileptic seizures or asymptomatic EEG anomalies. The seizures vary in terms of age of onset, signs and symptoms, and severity [38, 39]. Brain imaging may reveal ventricular or citerna magna enlargement, frontal lobe calcification, partial cerebellar agenesis, and ‘molar tooth sign’ [38, 39]. One SMS subject with Moyamoya disease has also been described [40]. In addition, the volume of the insulo-lenticular gray matter may be reduced bilaterally in persons with SMS [37].

## Neurocognitive disorders

Virtually all SMS children show a more-or-less pronounced speech delay, with potentially substantial lag (until age 7) [20]. Oral expression is often difficult, although comprehension skills are better. This discrepancy probably exacerbates behavioral disorders and seems to be quite typical of the syndrome. Developing the different modalities of language is thus a treatment priority.

Studies on the specific cognitive features of SMS persons are scarce. It seems that most patients show moderate intellectual deficiency, with an IQ between 40 and 54 [41, 42]. However, in Osório et al.'s (2012) study on a group of nine children, two had only slight intellectual deficiency and one, whose IQ was at the low end of the general population mean, did not fall into the deficient category at all [43]. SMS subjects' intelligence thus covers a wide range of levels [41–43], and their difficulties seem to increase with the extent of the deletion [44]. In our experience, the gap between SMS children and other children (especially regarding speech delay) often widens starting at the age of 3, when more specific cognitive disorders set in. However, hyperactivity and attention disorders worsen the child's problems at school, although long-term memory and perceptual abilities are relatively well preserved. By contrast, there is often a more pronounced deficit in short-term memory, sequential information processing, and visuomotor, attentional and executive abilities. There is apparently no premature age-related cognitive decline in this syndrome [43].

These findings confirm the importance of proposing individualized neuropsychological assessments, and suggest that the capacities of these patients may be underestimated. What's more, the exact impact of therapy involving early stimulation of neurocognitive functions has not been documented yet. Their difficulty fitting in socially is not linked solely to the cognitive phenotype. Behavioral and sleep disorders also have a deleterious impact on the quality of life of the patients, their family, and all the people who support them.

## Behavioral disorders

**Fig. 2:**
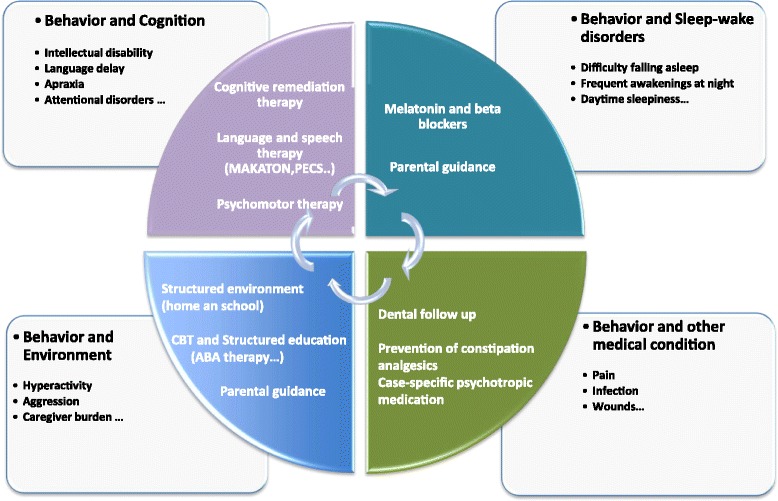
Proposal of a multimodal management of the behavioral disorders in SMS. Treatment of SMS is complex and includes: geneticists, neuropediatricians/neurologists, somnologists, developmental and behavioral pediatricians, psychiatrists, speech and language therapists, neuropsychologists, psychomotor therapists

Poor social integration in SMS adults is driven by intellectual deficiency but also by persistent chronic behavioral disturbance. Thus, an appropriate strategy should be started early in childhood and should integrate the different behavioral modalities (Fig. 2).

### Context of behavioral disorders

In our experience, behavioral disorders often appear with school or group socialization. They often come in the form of self-aggressive acts like biting, head banging, and picking at wounds, which then become chronic. In our experience, behavioral symptoms are variable in terms of severity: from mild phenotype (head banging and finger biting) to severe injuries (recurrent insertion of pointed objects in soft tissues, third-degree burns, severe aggression of close relatives …). Stereotypies are common, especially self-hugging and the tendency to keep one's hands in one's mouth which is probably the most specific in SMS and is usually accompanied by hand and fingers biting. Other less common stereotypies include licking the index finger and mechanically turning the pages of a book (“lick and flip”), body rocking, gritting one's teeth, etc. [6, 45, 46]. During this early period, SMS children frequently have temper tantrums and show impulsiveness, clastic behavior, and abrupt changes in attitude. Change-related anxiety is great, and their ability to adapt to the surrounding environment is limited [45, 46].

An important point is that among all the behavior disorders encountered in SMS, aggressive behaviors seem almost constant [47–50]. For example in a cohort of 32 SMS, the prevalence data was of 96.9 % for self-injurious behaviors and 87.5 % for physical aggression. This appears to be a specificity of the SMS, with significantly higher rates of aggression and destructive behaviors in SMS people in comparison to patients with intellectual deficiency of mixed origin [50]. Therefore aggression and destruction seem to constitute a classical phenotype in SMS. Indeed, other neurodevelopmental disorders, such as Rett or X fragile syndromes, inconstantly exhibit aggressiveness. Among self-injurious behaviors, fingers and hand biting are very evocative of SMS, especially in a child with development delay and sleep disorders. Aggressiveness directed toward others can also be seen. SMS children often seek for adult attention and seem to have low interest in other children [45, 51]. Aggression toward other, especially directed to close relatives, can be either verbal or physical. In our experience, behavioral disturbances are not always impulsive and can even be planned, which is disconcerting for the entourage and may be another specificity of this syndrome. Indeed, lack of expressive language, as observed in other neurodevelopmental disorders, is an aggravating factor. But it is not causal: impulsivity, aggression and hyperactivity may often increase after a few years at school despite the improvement of communication.

SMS patients may fulfill DSM-5 criteria for specific diagnoses in case of autism spectrum disorders and/or for hyperactivity and attention disorders [52]. This observation raises the question of the use of methylphenidate in those cases (for its effect on hyperactivity and as a wake-promoting agent in patients with comorbid sleep disturbance [29, 53, 54]. Anxiety and major depressive disorders can also be observed. It is to note that aggressiveness is not strongly linked to the presence of autism features or of hyperactivity. It seems mainly correlated to attention disorders but that does not mean a causal effect between these two features [50].

#### Behavior and sleep disorders

Maladaptive behaviors are often exacerbated by irregular sleeping patterns. Sleep disorders are common in neurodevelopmental disorders. For example 32 % of patients with fragile X syndrome had at least one indication of abnormal sleep in a parental survey study [55]. Sleep disorders are also frequent in many other disorders such as Rett or Prader Willi syndrome for example. Studies do not always concur on the nature of sleep disturbances in these syndromes which are usually multi-factorial [56]. Sleep disorder in SMS syndrome are a particular case among neurodevelopmental disorders and therapeutic strategies follow those particularities. First, sleep/wake disorders are almost constant in the syndrome. They are intense with heavy consequences on the caregivers. Second, the link between SMS sleeps disorders and inverted melatonin secretion is clearly established. As underlined by Ann Smith, in the 7 th international American conference on Smith Magenis syndrome: when untreated, ‘sleep disorders are the biggest problem in SMS’. Diurnal secretion of melatonin is associated with ‘jet lag-like’ drowsiness and therefore plays a major role in daytime behavioral disorders, especially among the youngest individuals. This aspect is usually alleviated by the use of beta-blockers. Conversely, the absence of nocturnal melatonin is a causal factor of shortened, fragmented nighttime sleep [30, 57] supporting as well behavioral disorders. Actually sleep deprivation, even in healthy children, contribute to neurocognitive disorders and disruptive behaviors. For example it may increase hyperactivity and attentional disorders. Sleep deprivation interfere with memory consolidation -especially semantic memory- and increase anxiety [55, 58, 59]. Nevertheless, in SMS residual maladaptive behavior often persists despite the treatment of sleep disturbances, and tends to increase with age. Thus, restlessness and aggressiveness (directed at oneself and/or others) seem inherent to SMS syndrome.

#### Behavior and pain

Decreased sensitivity to pain is a common feature of SMS [20, 37]. However, its precise pathophysiology remains unknown. This phenomenon is generally considered as a reflection of an underlying peripheral neuropathy linked to the loss of the *PMP22* gene across the microdeletion. On the other hand, one functional MRI and H2O PET study suggests the involvement of the central nervous system, and more precisely of the insular cortex [37]. The contribution of this decreased sensitivity to pain to behavioral disturbances remains to be defined. As underlined by Boddaert et al., pathological conditions with reduced sensitivity to pain are not necessarily associated with self-injury [37]. On the other hand, a high threshold of pain may hide medical conditions, such as dental infection, that may support behavioral disturbances.

#### Behavior and neurocognition

Behavioral disorders are partly related to neurocognitive impairment. Speech delay especially may lead to intense temper tantrums. Difficulties understanding prohibitions and implicit notions may lead to maladaptive behavior. Similarly, sexual development during adolescence may be associated with specific behavioral disorders that require further studies.

#### Behavior and environment

The patient’s environment has a significant impact on behavior. An astute study by Taylor and al., suggests that SMS self-injurious behavior and aggressive/disruptive outbursts are often evoked by low levels of adult attention and lead to increased levels of attention following the behaviors [51]. In our experience, this kind of behavior is exacerbated when the children are interacting with their close relatives, especially their mother.

On the other hand, emotional impact of having a child with SMS and behavioral problems may in turn increases the disorders. It is noteworthy that one of the characteristics of the SMS is that sleep disorders are so deep that the family is usually exhausted which deepens the difficulty to face the behavioral disruptive disorders.

Suffering at school or in the institution may emerge from conflicts with other persons (students or teaching staff) or poor school performance. All those situations should be systematically identified and evaluated.

In adulthood, the complete clinical picture entails poor social adjustment, often ending in institutionalization, with symptoms tending to resist or escape treatment [29, 45].

## Treatment strategies to prevent behavioral disturbance

So far, as for many orphan diseases, no general consensus on the treatment of behavioral disorders in SMS has been reached, and there are no recommendations on the prescription of psychotropic drugs [54]. However, an optimal strategy should integrate all the parameters detailed in Fig. 2.

Psychiatric symptoms should be precisely identified to determine case-specific medication. The antipsychotic monotherapy is indicated in order to limit side effects. The use of clozapine seems of particular interest in SMS. If required, antipsychotic cotreatment may be superior to monotherapy.

The use of methylphenidate for hyperactivity may also require further evaluation, especially during adulthood. Nevertheless, behavior management is not limited to medication and treatment should be comprehensive and integrative.

## Behavior and sleep disorders

Because sleep disorders favors behavioral disturbances that may in turn increase sleep disruptive behavior, they should be treated as soon as they appear. For this reason, an annual evaluation seems of interest in SMS. The treatment has been proposed on the basis of the known inversion of melatonin secretion in SMS [30, 31]. Usual medication includes melatonin in the evening (in general, 2 to 6 mg of prolonged-release melatonin) and beta-blockers (such as Acebutolol, 10 mg/kg) in the morning [60]. No clinical trial testing the effectiveness of the various pharmacological regimens proposed for treatment has been published so far. Education of the parents is an important component for the regulation of sleep disorders (e.g. avoiding sleeping with the child, no invasive games or rituals during night wakings, etc.…). The exact frequency of sleep breathing disorders is unknown in SMS. The risk is probably higher than in the general population, especially because of frequent overweight/obesity and use of high posology of antipsychotic medication [2, 19–21]. Sleep breathing disorders should be evocated in case of daytime sleepiness resisting to beta blockers, especially in patients with android obesity and/or taking psychotropic drugs. In our experience, sleep disorders spontaneously improve in young adults but the reasons remain unclear. Thus, whenever possible, treatment interruption should be considered to assess the usefulness of continuing pharmacological intervention.

## Behavior and pain

When facing a recent increase of behavioral disorders, the practitioner should consider the possibility of an underlying medical condition. Optimal intervention requires the systematic research and treatment of pain, including inflammatory, dental, acute, chronic, premenstrual, visceral pain and headaches. In our experience, a dramatic increase of aggressive and/or self-injurious behaviors may only reveal severe transit disorders in SMS adults.

## Behavior and neurocognition

In general, language and speech therapies are a major stake in the early prevention of behavioral disorders, especially in case of language delay. In SMS, it should be initiated as soon as possible (by the age of 6–8 months) as a priority, using signs and symbols such as pictograms or the MAKATON method. A multimodal approach to communication is recommended because the main difficulties concern the expressive language [61, 62]. Language therapy is designed to help children gain access to oral language and limit the frustration due to their poor ability to express themselves. It relies among others on self-expression activities, and swallowing and tongue positioning exercises. Augmentative communication approaches are standard for children with severe expressive language delay/impairment. They may include eye tracking devices for children with special needs such as motor impairment. Its interest in SMS children, especially those with autism spectrum disorders and/or hyperactivity, requests further studies [63, 64]. Dyspraxia may require psychomotor therapy.

Neuropsychological assessment is useful in drawing up the overall picture of the child's skills. Knowing the full extent of their capacities is important since it helps the children improve their practice; it also enables practitioners to adapt the treatment to the child (e.g. with the use of a computer) and propose appropriate schooling. Activities requiring the use of visuospatial, sequential and coordination skills are also recommended. In association with pharmacological approaches, cognitive remediation is of peculiar interest in SMS. SMS cognitive profile is often heterogeneous with relatively preserved skills alongside specific deficits. Remediation helps develop adapted strategies to supply for impaired cognitive processes, which is paramount in developing the patient’s autonomy. Last but not least, deficits such as attention deficit or hyperactivity may induce conduct disorders [62, 65–67]. Thus, cognitive remediation may have a positive impact on SMS behavioral disorders and reduce the number of institutionalized SMS patients in the long term.

## Behavior and environment

At school, SMS children are easily distracted and hyperactive. Hence, small classes are more adapted because they offer a more structured, less distracting learning environment. Working with the teaching staff (especially specialized personnel) is of utmost importance. Because SMS children have difficulty adapting to change (due to low cognitive flexibility), they have to be warned and prepared in order to avoid anxiety-related manifestations of their behavioral problems. Children should develop in the most stable possible school environment – one that enhances their sense of security. Given that these children have trouble understanding sequential information, the use of a pictorial calendar and timer may help them form concrete mental images of passing time and daily schedule. SMS adolescents and young adults should be encouraged to achieve autonomy. Their academic and then occupational careers should be adapted to their competencies and guided by their results on recent neuropsychological assessments. Another key part of the treatment involves working with the family and providing parental guidance.

Given the fascination of SMS children for screens the use of computers and pads may be useful to reinforce their learnings. For fine motors skills example writing with a pen is harder in SMS because of fine motors skill disabilities and reading is also hard because of attentional disorders. Using a pad or a computer may help SMS of all ages to start to read and write.

Last but not least, regular psychiatric follow-up is paramount in the management of these patients In SMS, clinical practice suggests a benefit of interventions focusing on providing parent training and cognitive behavioral therapy (CBT). Bolstering social skills and managing challenging behaviors or sleep disturbances may improve social communication, language use, and potentially symptom severity. But the lack of consistent data limits our understanding of whether these interventions are linked to specific clinically meaningful changes in SMS functioning. Stress, anxiety and mood disorders are associated with faulty emotional regulation. A large proportion of persons with intellectual disabilities show these symptoms [68]. Aggression and physical violence could be enhanced by a poor control in emotional regulation [69]. This situation might explain the particularly high prevalence of challenging behavior in SMS people. For Whitaker, there seems to be some evidence that cognitively based emotions control treatment can be effective with people who have learning disabilities [70]. Mindfulness-Based Treatment has been adapted in Individuals with mild intellectual disabilities and physical and verbal aggression behaviors [71]. Its precise interest in SMS remains to be evaluated.

All these measures are not limited to children and should be carried on throughout the life of the patient.

## Summary

The prognosis of SMS patients today is closely linked to their behavioral manifestations. In adulthood, they sometimes require repeated hospitalizations in psychiatric wards, or may even have to be institutionalized. Treatment of SMS is complex and requires a multidisciplinary team including, among others, geneticists, neuropediatricians/neurologists, somnologists, developmental and behavioral pediatricians, psychiatrists and speech and language therapists. An optimal care plan includes treating sleep/wake rhythm disturbances and behavioral disorders. Another key part of the treatment involves working with the family and providing parental guidance. In any case, the emphasis should be on valorizing each individual's abilities in order to help them overcome their specific difficulties. Lastly, the issue of treating behavioral disorders related to syndromes is not limited to SMS, and the propositions developed in this paper extend to the large group of genetic disorders associated with neurobehavioral phenotypes.

## Constent

Written informed consent was obtained from the patient's guardian/parents/next of kin for the publication of this report and accompanying images.

## Abbreviations

SMS: Smith Magenis syndrome
RAI1: Retinoic Acid Induced 1
